# To Prevent Night-Sweats in Phthisis

**Published:** 1854-08

**Authors:** 


*To Prevent Night-sweats in Phthisis.*—Night-perspirations in the course of phthisis are often extremely annoying to the patient; they appear, also, to be simply debilitating, and unattended by any degree of collateral benefit. Some cases which were carefully noted by Mr. Hutchinson, the Clinical Assistant at the City Hospital for Chest Diseases, with a view to the determination of that question, appeared to show that they may be artificially checked, not only with impunity, but with great benefit. The patients who were so treated, and who, in the course of a week or a fortnight, got quite rid of sweatings, which for months had been profuse, thought themselves much better, and did not complain of increase, either as regards the expectoration, the feverishness, or the sense of stuffing in the chest. Under the usual treatment of phthisis, (full diet, cod-liver oil, and tonics,) the tendency to night-perspirations often ceases spontaneously. If it becomes desirable to expedite the process, it may be done by the sesquichloride of iron, the mineral acids, or, best of all, by the gallic acid. The following is the prescription for a night draught containing the latter:—

℞ Acidi gallici. gr. vij.;

Morph. acet. gr. $\frac{1}{8}$;

Alcohol q. s. (a few drops);

Syr. tolutan. ʒss;

Aquæ ℥j.

The night-pill, as we find in the Pharmacopœia of the Brompton Hospital for consumption, is—

℞ Acid. gallic. gr. v.;

Morph. hydrochl. gr. $\frac{1}{8}$;

Mist. acac. q. s. Ft. pil. ij,

It is also of advantage to adopt an astringent regimen as far as convenient.

The patient should be directed to sleep on a mattress, alone, and not heavily clothed; he should wear no flannel in bed; as dry a diet should be taken as conveniently can be borne, and fluid should be especially avoided in the latter half of the day, none whatever being allowed later than several hours before bed-time.—*London Medical Times.*

